# Dietary Manipulation on Gut Microbiome in Patients with Diabetes and Colorectal cancer

**DOI:** 10.1007/s13668-025-00667-8

**Published:** 2025-06-03

**Authors:** Natural H. S Chu

**Affiliations:** Department of Medicine and Therapeutics, Prince of Wales Hospital, The Chinese University of Hong Kong, Hong Kong SAR, China

**Keywords:** Fermentable carbohydrates, Fermentable protein, Red meat, Specific intestinal microbiome, Diabetes, Colorectal cancer

## Abstract

**Purpose of review:**

This review aims to investigate the relationship between dietary changes and the microbiome in patients with diabetes and colorectal cancer (CRC). The link between CRC and type 2 diabetes is momentous, as individuals with diabetes have a 40–60% higher risk of developing CRC and often experience lower survival rates. However, limited studies explore how diabetes may contribute to the progression to CRC through changes in the microbiome. By clarifying these connections, this review summarizes mechanisms in type 2 diabetes and CRC through microbiota pathways, presenting evidence from clinical trials regarding nutritional interventions for treating both conditions. We will focus on how nutritional components can alter the gut microbiome, highlighting the potential role of nutritional adjustments as adjuvant therapy for patients with diabetes who are facing precancerous or cancerous conditions.

**Recent Findings:**

There is growing evidence about the interactions between the microbiome and the causes of diabetes and CRC. Both conditions are characterised by changes in the gut microbiome, known as dysbiosis, which involves alterations in specific bacteria, such as *Bifidobacterium, Bacteroides, Akkermansia, Faecalibacterium, Ruminococcus*, and *Fusobacterium*. It is important to consider dietary modifications to address dysbiosis, malnutrition, glycemic variability, and inflammation underlying these conditions.

**Summary:**

Consuming a higher amount of fermentable carbohydrates alongside a lower amount of fermentable proteins can positively influence the microenvironment that regulates insulin secretion and bile acids, as well as an increase in short-chain fatty acids. This may be beneficial for patients with diabetes and CRC. However, it is also important to consider potential interactions between food and medication as well as gastrointestinal tolerability.

## Introduction

Colorectal cancer (CRC) is the third most common cancer worldwide. Global cancer statistics indicate that it is the fourth leading cause of cancer mortality and imposes an enormous burden on society worldwide [1]. Exogenous and endogenous factors, such as genetic predisposition, lifestyle (diet and physical activity), medications (aspirin etc.), and environmental influences, contribute to the characteristics of neoplastic cells and non-transformed non-neoplastic cells within the tumor microenvironment [2, 3]. The expansion of clones with somatic mutations in non-neoplastic tissues is particularly notable in the context of aging or chronic inflammation [4], while diabetes is a condition characterized by systemic inflammation and also influenced by the aforementioned factors [5].

An analysis of 80,193 gastrointestinal cancers from five European and three Asian countries revealed that the prevalence of diabetes was greater in patients with colon (15.5%) or rectal (15.3%) cancer than in those with other gastrointestinal cancers [6]. In a recent comprehensive systematic review and meta-analysis that reported CRC incidence and risk in prediabetic cohorts, the odds ratio of developing CRC was 16% greater in patients with prediabetes than in those with normal glucose regulation (NGR) [7]. Hyperinsulinemia, hyperglycemia, and type 2 diabetes have been associated with a 40–60% increased risk of colorectal cancer [8–10], with lower overall rates of survival in diabetic patients with CRC, irrespective of body mass index [11].

## Relationships and Mechanisms Between Colorectal Cancer and Diabetes

The pathogenic link between diabetes and colorectal cancer may occur through various mechanisms, from hyperinsulinaemia, hyperglycemia to increased oxidative stress and inflammation from endogenous and exogenous sources, including the alterations of gut microbiome [12].

Hyperinsulinemia is a condition of increased levels of circulating insulin in the blood, ultimately increasing the availability of insulin-like growth factor (IGF)-I to the IGF-I receptor, which is crucial in the development of colorectal cancer [13–15]. IGF-1 and insulin activate the phosphatidylinositol-4,5-bisphosphonate 3-kinase (PI3K) signalling pathway, which regulates the expression of fatty acid synthase (FASN), leading to increased lipogenesis early in carcinogenesis [16, 17]. Patients undergoing insulin therapy may experience a heightened risk of inducing carcinogenesis in normal colorectal epithelial cells, as demonstrated in vivo [18]. High insulin levels stimulate the production of IGF-1 by liver cells after insulin binds to its receptor, whereas IGF-1 can also bind to insulin-like growth factor binding protein 3 (IGFBP-3), which is associated with a greater risk of colorectal cancer [19]. Another feature associated with diabetes is hyperglycemia, which provides direct sources of blood glucose. When the expressions of glucose transporters (GLUT), including GLUT 1 and GLUT 3, are upregulated by oncogenes in tumor cells [20], glucose fuels energy for cell growth and proliferation. Moreover, hyperglycemia leads to the accumulation of advanced glycation end products (AGEs), which stimulate chronic inflammation and oxidative stress [21].

This introduces another possible mechanism involved in the complex aetiology of diabetes and colorectal cancer, highlighting the multifactorial risk assessment that identifies the synergistic effects of oxidative stress and inflammation as critical components for the development of both diseases. Chronic hyperinsulinemia and hyperglycemia can contribute to visceral adiposity, which produces inflammatory factors, free fatty acids, adipokines, proangiogenic factors, and components of the extracellular matrix [22–24]. For example, studies in diabetic animal models show elevated levels of inflammatory cytokines, such as interleukin (IL)-1β, as well as changes in the activity of enzymes like NADPH oxidase (NOX)4 in colon tissues following the induction of colorectal cancer [25].

Dysbiosis, a disruption of the gut microbiome, has also been linked to the risk of both diabetes and CRC [26, 27]. Patients with type 2 diabetes and CRC had lower microbial α diversity than healthy controls did [28, 29], overlapping with changes in *Bifidobacterium, Bacteroides*,* Akkermansia, Faecalibacterium, Ruminococcus*, and *Fusobacterium* [30, 31]. Several bacterial species have been shown to exhibit proinflammatory and procarcinogenic properties, which could consequently have an impact on colorectal carcinogenesis [32–34]. For example, *Fusobacterium nucleatum* has been shown to increase inflammatory cytokines, leading to systemic damage to cell functions in both diseases [35, 36]. *Fusobacterium nucleatum* attach to cancer cells via cell surface attachment proteins, activating the PI3k-Akt pathway, which controls cell growth and leads to cell proliferation [37, 38]. *Akkermansia muciniphila* stimulates mucus production and regulates immunomodulatory activity, protecting against infection caused by pathogens in people with diabetes [39]. Some studies also revealed that a decreased abundance of *Akkermansia muciniphila* was associated with severe symptoms of CRC [40]. Nonetheless, there is ongoing debate regarding the role of *Akkermansia muciniphila* in bacterial overgrowth associated with colorectal carcinogenesis [41, 42], highlighting the importance of maintaining a delicate balance of microbial homeostasis and health [43]. Moreover, impaired conversion of primary bile acids to secondary bile acids can lead to increased oxidative stress and DNA damage [44], with an increased abundance of Enterobacteriaceae and a lower abundance of 7α-dehydroxylating bacteria (e.g., *Clostridium* spp.) [45, 46]. Bile acids have emerged as pleiotropic signalling molecules that mediate intestinal tumorigenesis and inflammation [46]. Genotypes of species and disease states could partly explain this discrepancy in the evaluation of the feasibility of microbiota-based therapies for CRC [47]. Together, these elements create a tumor-supporting microenvironment in patients with diabetes.

**Fig. 1:**
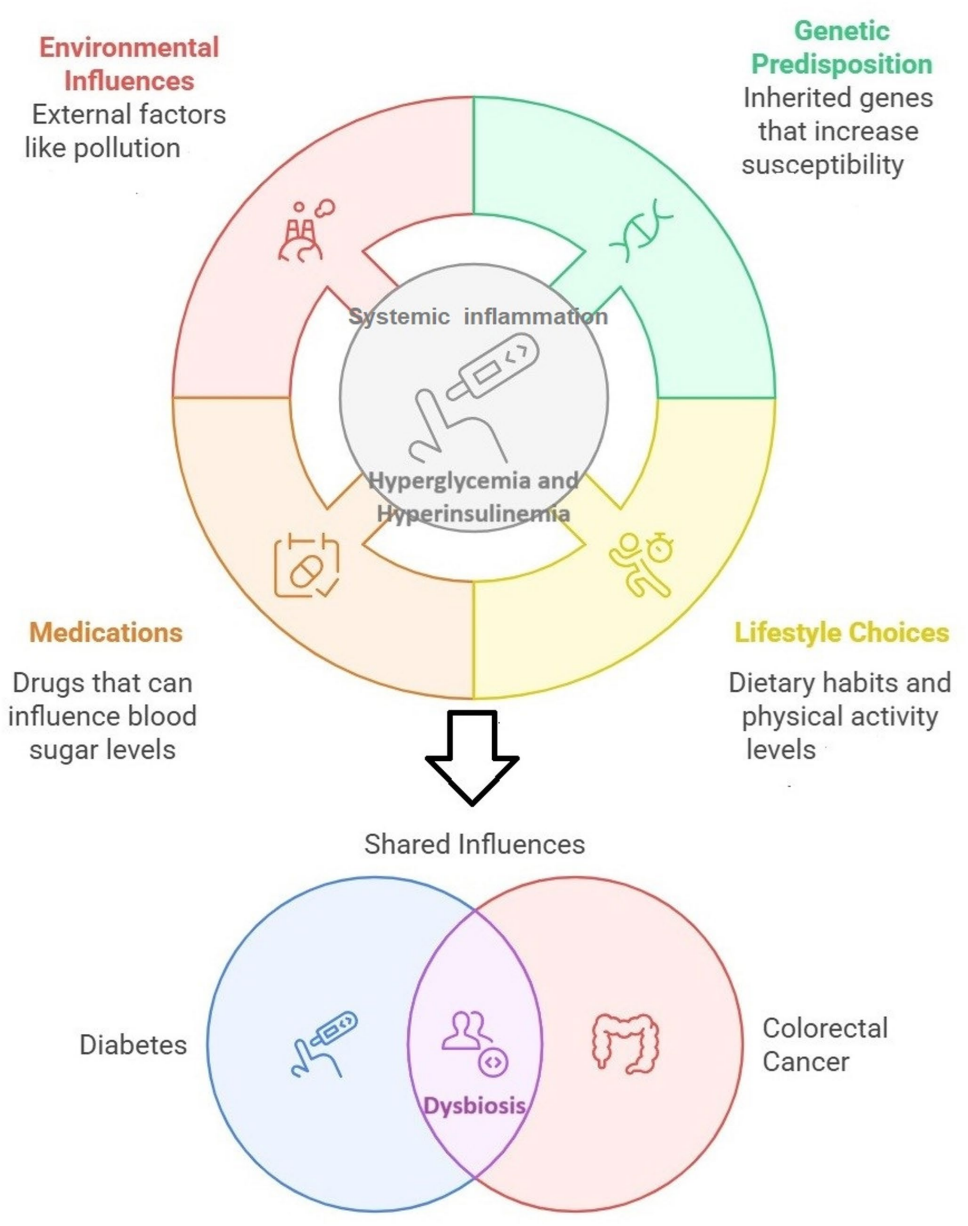
Pathophysiological mechanisms between diabetes and colorectal cancer

The pathophysiological mechanisms between obesity and CRC are shown in Fig. 1, whereas diet is one of the important factors directly linked to both microbial and immune mechanisms of colorectal cancer and diabetes.

Crosstalk between the microbiome and its metabolites has been well discussed in the context of CRC or diabetes; however, effective dietary intervention for both individuals with diabetes and CRC is limited, and improving cancer outcomes in survivors are called from those demographic data [48, 49]. This review first examines the mechanisms that link type 2 diabetes and CRC through microbial pathways. It then discusses the evidence from clinical trials regarding nutritional interventions aimed at managing both conditions. Finally, the review explores the interaction between key macronutrients and fiber with the gut microbiome, with a particular focus on the intake of fermentable carbohydrates and proteins. This highlights the potential of nutritional modifications as a complementary therapy for diabetes patients who are facing precancerous or cancerous conditions.

### Nutritional Interventions for Diabetes and CRC

Research has suggested that low-glycaemic index (GI) diets reduce glycated proteins 7.4% more than high-GI diets do [50] in 14 randomised crossover or parallel experimental designs for diabetes patients, but another study comparing high-legume and low-GI diets suggested that no significant differences related to the glycaemic index were detected in at risk colorectal patients [51]. Recent studies have shown that Mediterranean diets improve postprandial glucose and insulin responses on diabetic patients [52]. A meta-analysis of epidemiological studies revealed an inverse association between Mediterranean diet adherence and CRC incidence [53]. Nevertheless, greater interpersonal variation depends on food patterns and interactions with the gut microbiome [52, 54]. There is limited evidence concerning the specific targeting of the gut microbiome through changes in the quality and quantity of the diet.

Research on the deregulation of the intestinal microbiota and its association with colorectal carcinogenesis has been thoroughly reviewed. The primary source of this review was the PubMed database, with additional articles accessed through the University Library Catalogues, MEDLINE, and Google Scholar. The search terms used were “colorectal cancer”, “gut microbiota”, and “diet”. The criteria for inclusion in this literature review were that the articles had to be full text, written in English, and published from 2011 to the present. In total, 24 articles met the search criteria. Four articles were published as protocols, two were unrelated to colorectal cancer, seven did not involve changes in the microbiome, and one was a cross-sectional study. Finally, we thoroughly reviewed 10 clinical trials, as detailed in Table 1.

**Table 1:** Proposed model for clinical trials on diet, the Microbiome and CRC

Author	Year	Subjects	Diet	Study type	Period	Outcomes
Zhang et al.[55]	2023	55 people with overweight and obese patients with a history of colorectal polyps or cancer	Usual diet without beans (control) or with a daily cup of study beans (intervention)	Randomised crossover design	8-week periods	↑*Faecalibacterium, Eubacterium* and *Bifidobacterium.* ↑*pipecolic acid* and *↓indole* in the intervention group
Farsi et al.[56]	2023	20 healthy male adults	240 g/day red and processed meat or Mycoprotein (nonmeat protein from fungus) for 2 weeks	Investigator-blind, randomised, crossover dietary intervention trial	2-week periods	↑*Lactobacilli*,* Roseburia*,* Akkermansia* and SCFA *↓*faecal genotoxicity and nitroso compounds
Byrd et al.[57]	2022	170 people adhering to the intervention and 198 people as a comparable control arm	A high-fibre, high-fruit and vegetable, and low-fat diet	Randomised, controlled trial	4 years	Higher circulating bile acids (glycochenodeoxycholic acid and glycocholic acid) were associated with baseline adenoma status and *Bacteroides.* However, no significant differences in bile acids were altered by the intervention.
So et al. [58]	2021	39 people with a high risk of CRC	30 g of rice bran (intervention) vs. 30 g of rice powder (control)	A double-blinded, randomised, placebo-controlled trial	24 weeks	*↑ Lactobacillus* and *Bifidobacteria* in the intervention arm than the control
McCann et al. [59]	2021	252 healthy, postmenopausal women	10 g/d ground flaxseed into their usual diet (intervention) or maintain their usual diet (control)	Randomised, crossover trial	6 weeks	CRC-related microbiome were reduced after the intervention, such as *Fusobacteria Odoribacteria*, and *Pyramidobacter.*
Griffin et al.[60]	2019	115 people at increased risk of CRC	Mediterranean diet intervention	Single-arm dietary intervention	6 months	There were no significant changes in plasma trimethylamine N-oxide (TMAO), but TMAO was negatively and modestly correlated with *Akkermansia muciniphila.*
Djuric et al.[61]	2018	93 people at increased risk of CRC	Either a Mediterranean diet or a Healthy Eating diet	Randomised dietary intervention trial	6 months	The carotenoid intakes increased in both diets similarly, and the microbiome had no differences. *Bacteroides*,* Roseburia*, and *Blautia* differed in tertile of total serum carotenoid concentration.
Eid et al.[62]	2015	22 healthy human volunteers	50 g/day of dates vs. maltodextrin-dextrose (control)	Randomised, controlled, crossover trial	21 days	↓stool ammonia concentration and genotoxicity in human faecal water, but not changes in the microbiota and SCFAs.
Le et al.[63]	2015	23 healthy volunteers	300 g/d of cooked red meat without or with 40 g/d of butylated high-amylose maize starch (HAMSB)	Randomised crossover design	4-week periods	Epithelial proliferation was lowered in the HAMSB diet, and the excretion of SCFA increased by over 20%, increasing the absolute abundances of the *Clostridium coccoides* group.
Russell et al.[64]	2011	17 obese men	High protein and moderate carbohydrate diet vs. high protein and low carbohydrate diet	Crossover design	4 weeks	A high-protein and low-carbohydrate diet significantly decreases faecal cancer-protective metabolites (↓SCFAs) and increases concentrations of hazardous metabolites. ↓*Roseburia/Eubacterium rectale* group

On the basis of the above clinical studies on CRC, a plant-based diet achieved better outcomes in promoting beneficial bacteria, including *Faecalibacterium, Eubacterium*,* Bifidobacterium*,* Lactobacilli*,* Roseburia*, and *Akkermansia.* It also influences the CRC-related microbiome, *Fusobacteria*,* Odoribacteria* and *Pyramidobacter*, as well as microbial metabolites, such as short-chain fatty acids (SCFAs), bile acids and the stool ammonia concentration. These microbiomes and their derived metabolites have been discussed previously in relation to the etiopathogenesis of both diabetes and CRC.

In a multicenter Italian case-control study including 1,953 histologically confirmed colorectal cancer cases, a diabetes risk reduction diet rich in vegetables, grains, coffee, nuts and a high polyunsaturated/saturated fat ratio was inversely related to colorectal cancer risk [65]. In another randomised, double-blind, controlled trial involving 35 patients with CRC, the intervention group consumed 900 mg of pomegranate extract daily before surgery. MicroRNAs were measured from malignant and normal colon biopsies as CRC biomarkers. The intake of pomegranate extract significantly altered the levels of miR-1249, miR-92b-5p, miR-765, miR-496, and miR-646 in malignant tissues, potentially reducing the burden of rectal polyps and cellular proliferation [66]. Notably, these food items are more likely to impact the gut microbiome than to exert antioxidant effects [67]. The clinical evidence supporting the contribution of dietary polyphenols to the prevention or reduction of CRC is still under debate [68]. Like polyphenols, there was no association between the intake of individual or total carotenoids and the risk of CRC overall or by anatomic subsite [67]. Therefore, the next section will focus on the interaction between fermentable nutrients and the gut microbiome.

## Mechanisms of Fermentable Carbohydrates and Proteins on Diabetes and CRC

Precision nutrition, which is a personalised nutritional approach based on individual characteristics, has shown promise in directly impacting the composition of the gut microbiome [69]. Most cancer patients have negative consequences from clinical treatment, such as chemotherapy. Malnutrition is associated with poorer prognosis in cancer patients [70], and dietary therapy has shown promising efficacy in improving serum albumin and prealbumin levels in colorectal cancer patients undergoing chemotherapy [71] and decreasing nutritional deterioration [72]. However, high-calorie and protein beverages are recommended for cancer patients [73] to maintain body weight and are easily consumed by substituting typical meals. A high-protein diet significantly decreases fecal cancer-protective metabolites and increases the concentrations of hazardous metabolites [64]. Modifying the gut microbiota as an adjuvant therapy for cancer patients reduces inflammation and the expression of cell proliferation markers in colon tissues [74].

Research has suggested that various fermentable carbohydrates can impact the gut microbiome and potentially aid in the treatment of metabolic diseases. This includes the impact on several microbiomes, such as *Butyricicoccus, Akkermansia*, and *Phascolarctobacterium*, and the possible mechanism of SCFA induction and reduction in primary bile acids [54]. However, habitual diets are complex and involve different nutritional components, such as protein and fats. While the microbiome primarily digests carbohydrates, current clinical practice focuses on increasing protein intake, especially in patients with diabetes, to manage conditions such as cachexia and improve glycemic control in patients with CRC [75, 76]. It is worth noting that excess amino acids may overactivate the mammalian target of rapamycin complex 1, resulting in desensitisation of the insulin receptor substrate and reducing insulin-mediated glucose uptake [77].

**Fig. 2:**
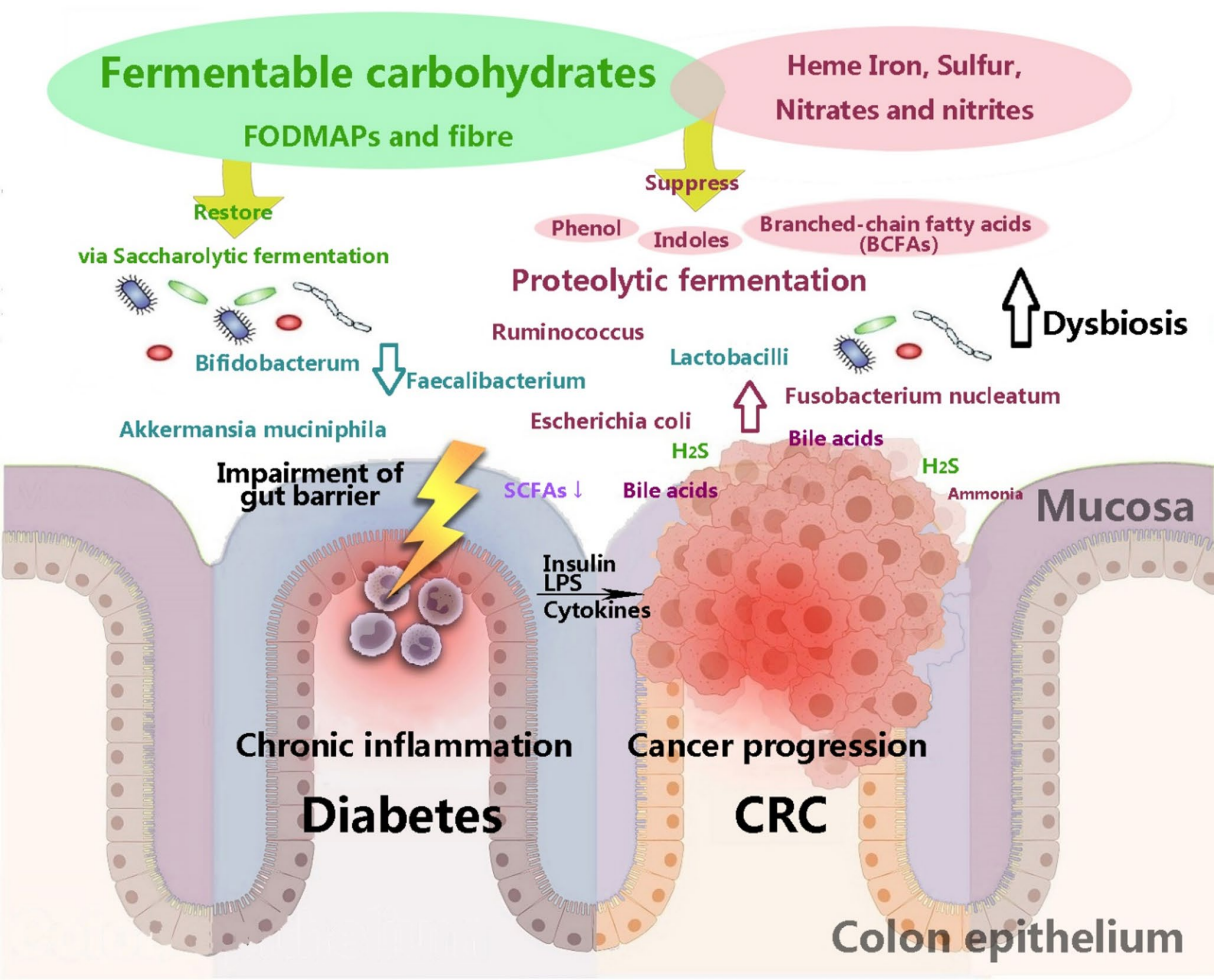
The possible mechanism of increased fermentable carbohydrates and reduced fermentable protein in patients with diabetes and colorectal cancer. ↓ indicates reductions, whereas ↑ indicates increased abundance. Abbreviations: CRC: colorectal cancer, LPS: lipopolysaccharides, SCFAs: short-chain fatty acids, H_2_S: hydrogen sulfide

Furthermore, consuming more protein may negatively impact the progression to CRC. When proteins are broken down through fermentation, they produce branched-chain fatty acids (BCFAs) as well as potentially harmful byproducts such as ammonia, indoles, and phenols [78]. Our gut microbiome has a process for breaking down amino acids that conserves more energy than their breakdown. Therefore, the gut microbiota preferentially consumes carbohydrates over proteins [79]. Nevertheless, the current report has shown that patients with CRC have reduced microbial diversity and a shift from the use of dietary carbohydrates to the breakdown of amino acids [80, 81]. As a result, the interaction between fermentable carbohydrates and protein in the context of diabetes and CRC is an important topic for discussion owing to their potential impact. They involved in modifying the gut microbiome to achieve glycemic control and anti-inflammatory effects in patients with diabetes and CRC, as illustrated in Fig. 2.

In the following sections, the role of diet, focusing on fermentable carbohydrates and proteins, is detailed:

## Dietary Fat

Current prospective cohort studies have not revealed a direct link between fat intake and the risk of both diabetes and CRC [82–84]. The main objective of dietary fat for people with diabetes is to limit saturated fat and dietary cholesterol intake to avoid energy over-absorption [85]. Recent findings suggest that eicosapentaenoic acid-free fatty acid (EPA‐FFA) can reduce polyp formation and growth in models of familial adenomatous polyposis, thus preventing inflammation in CRC and leading to an increase in *Lactobacillus* species in the gut [86]. A study involving 217 healthy individuals who consumed a high-fat diet revealed a significant increase in *Alistipes* and *Bacteroides* and a decrease in *Faecalibacterium*. The concentration of total SCFAs was also significantly decreased in the high-fat diet group, and there was an increase in plasma proinflammatory factors after the intervention [87]. However, a high-fat diet is not commonly found in the typical diets of patients suffering from diabetes and CRC. While there is no overall positive or negative correlation between colorectal cancer risk and total or subclass fat intake, the use of oils with a high smoke point is recommended to reduce the formation of free fatty acids from the hydrolysis of triglycerides and decrease the production of AGEs [88].

## Dietary Protein

### Fermentable Proteins for Diabetes and CRC

The digestibility of food may be affected by its protein source and how it is processed. Even when highly digestible proteins are consumed, a significant portion may not be absorbed and instead fermented in the large intestine. These fermentable proteins could have adverse effects on the gut microbiota and lead to chronic inflammation [89]. The impact of heat or processing on protein might also affect its digestibility. For example, the digestibility of casein was reduced by 90% after being thermolyzed for one hour [90]. A similar reduction in protein quality was observed when the thermolysis of soy and egg proteins decreased [91]. Hence, the fermentability of protein relies on its digestibility, which is influenced by the cooking time and temperature. When higher levels of fermentable protein reach the large intestine, they can interact with the gut microbiome and cause dysbiosis. It is important to consider the following elements in fermentable protein:

### Heme Iron from Red Meat

Numerous observational and epidemiological studies have demonstrated a connection between high consumption of red and processed meat and an elevated risk of colorectal cancer and diabetes [92–94]. Consumption of red and processed meats is associated with higher levels of high-sensitivity C-reactive protein [95], which induce the systemic inflammation for both diabetes and CRC. Meats containing high levels of haemoglobin due to preservation methods such as smoking, curing, salting, or chemical additives can be detrimental to colon health and may result in the production of potentially carcinogenic compounds, including heterocyclic aromatic amines (HCAs), which are considered potential carcinogens. Additionally, certain bacterial species found at higher intakes of red meat, such as *Escherichia coli* and *Bacteroides fragilis*, have been associated with genotoxic potential [96, 97].

The carcinogenicity of heme iron from red and processed meat is also linked to the formation of genotoxic N-nitroso compounds (NOCs), which are potent carcinogens in the gastrointestinal tract [98]. Owing to redox reactions, reactive oxygen species (ROS) and lipid peroxidation products (LPOs) exert genotoxic effects by alkylating agents (N-alkyl-NOCs) throughout the metabolic process and induce transitions of DNA bases from GCs to ATs in human colonocytes after high red meat consumption [99]. Polycyclic aromatic hydrocarbons (PAHs) and HACs are generated during high-temperature cooking and contribute to carcinogenesis. These chemicals also induce the infiltration of myeloid cells into the colorectal mucosa, accompanied by an increased level of cyclooxygenase-2 (COX-2)-positive cells, as demonstrated in the in Vivo experiments [100]. In an animal model of colitis or colitis-associated adenoma, supplementation with heme reduced α diversity, leading to a reduction in the SCFA-producing phylum Firmicutes and an increase in the pathogenic phylum Proteobacteria, particularly Enterobacteriaceae [101]. However, eliminating red meat depletes iron storage, and there is inadequate production of heme in the body. A decrease of 50 g/d in processed meat consumption lowers the total number of colorectal cancer cases by approximately 20%, and it is also suggested that the consumption of red meat should be less than 70 g/week [102]. For example, 70 g of cooked beef per week is equivalent to 90 g of cooked chicken per day in terms of heme iron content [103].

## Sulfur Elements from Methionine and Cysteine

Sulfur is present in amino acids from various food groups and is also used as a preservative in processed meat. It plays a significant role in the metabolism of tumor cells. Sulfur compounds, such as those in sulfur-containing amino acids, methionine and cysteine, are broken down into hydrogen sulfide (H_2_S), sulfite (SO_3_^2−^), thiosulfate (S_2_O_3_^2−^), and sulfate (SO_4_^2−^) in the human gut [104]. The production of H_2_S, a crucial component of this process, is attributed to both endogenous enzymes (including cystathionine β-synthase (CBS), cystathionine γ-layse (CSE), and 3-mercaptopyruvate sulfurtransferase (3-MST)) and exogenous sulfate/sulfur-reducing bacteria via microbial sulfidogenesis, including changes in *Bilophila, Desulfovibrio, Desulfomicrobium*, and *Fusobacterium* [105]. Elevated levels of H_2_S impact both oxidative and glycolytic metabolism in tumor cells, leading to DNA damage, epithelial hyperproliferation, and inflammation [106]. For example, *Bilophila wadsworthia* is highly correlated with colorectal cancer and increased systemic inflammation and abundance at the onset of latent autoimmune diabetes in adults (LADA) [107–109]. Other sulfide-reducing bacteria, such as *Atopobium parvulum* and *Actinomycosis odontolyticus*, are also linked to multiple polypoid adenomas and intramucosal carcinomas in the colon and rectum [110].

However, low doses of H_2_S are swiftly detoxified into thiosulfate by the mitochondria of colonic epithelial cells, and the small intestine is efficient at absorbing sulfates at lower dietary intakes [111, 112]. This detoxification process helps to protect cells from the potentially harmful effects of H₂S [113]. In contrast to the external production of H_2_S from the microbiome, the endogenous production of H₂S in islet β cells, the liver, adipose, skeletal muscles, and the hypothalamus, which regulates local and systemic glucose metabolism, has a potential role in diabetes [114]. Another source of allyl sulfur components is naturally occurring foods such as garlic, onion, and sulfur-containing glycosides, mainly glucosinolates, which are present in cruciferous vegetables, as indicated in Table 2. These vegetables appear to have a protective effect on colorectal cancer. For example, in case‒control studies, garlic was linked to an approximately 37% reduction in colorectal cancer risk [115]. This protective effect can be attributed to the greater amounts of short-chain fermentable carbohydrates found in Brassicaceae family plants (Table 2) (discussed in a later section), which are associated with lower body fat and higher insulin sensitivity [116]. Low sulfur intake was linked to a change in nitrate concentrations [117], another potential factor influencing CRC development through the formation of nitrosamines.

**Table 2:** List of sulfur- and nitrate-containing foods

Nutrients	Food sources	
Sulfates & sulfites	Food additives	Bread, baked goods Dried fruits, nuts Processed vegetables Preserved meat and fermented drink
	Brassicaceae family plants	Cauliflower, broccoli, Brussels sprouts garlic, onion
Nitrates & nitrites	As food additives	Salami, ham, bacon
	Natural food sources	Beetroot, cabbage, lettuce, turnip
	Tap water	Drinking water, water for cooking

## Nitrate and Nitrite

Nitrate and nitrate present in the aquatic environment due to human activities, but more than 80% of their occurrence is natural and is found in foods (Table 2). A controlled-feeding study was conducted to compare 4 different diets, including phytochemical-enriched conventional processed and/or low-nitrite processed meat or conventional processed meat or diets high in poultry, each for two weeks in 63 healthy subjects. After these diets were completed, an additional nitrate-enriched water phase lasting 1 week was added to these 4 diets in a random fashion. Nitrate-enriched water significantly reduces oral microbial activity but does not affect the gut microbiome across diets [118]. Like endogenously produced H_2_S, the inorganic anions nitrate (NO3-) and nitrite (NO2-), which originate from dietary nitrate or nitrite endogenously in our bodies, act as pleiotropic signalling molecules that improve pancreatic islet function [119]. Excess intake of nitrate or nitrite from water or the diet leads to the endogenous synthesis of NOCs and is linked to an increased risk of GI cancer [120]. The World Health Organisation (WHO) considers 50 mg/L to be a safe standard for nitrate and nitrite levels in drinking water. However, several studies have indicated that consuming 4 to 5 mg/d of waterborne nitrate may increase the risk of colon cancer, whether through drinking water or the cooking process [121, 122]. Additionally, nitrate and nitrite from food additives in processed meats increase the risk more than those from natural foods do, while natural nitrates have a protective effect due to their nitrosation inhibitors [123].

### Tryptophan

In addition to these critical protein elements, tryptophan is an essential amino acid that the host cannot endogenously synthesise. Therefore, dietary tryptophan is obtained through meat, dairy, and seeds, and approximately 4–6% of dietary tryptophan enters the colon and reacts with the gut microbiome [124]. The gut microbiome plays a crucial role in tryptophan metabolism, which is often overlooked. Interestingly, circulating tryptophan is inversely associated with diabetes and CRC because the inhibition of indoleamine 2,3-dioxygenase 1 (IDO1) increases antitumour immunity [125, 126]. In CRC patients, dysbiosis with increasing *Enterococcus faecalis* and *Escherichia coli* influences the production of the intestinal inflammatory signalling molecules IFN-γ and IL-4, converting the expression of IDO1 and altering tryptophan metabolism [127].

Additionally, *Fusobacterium nucleatum*, a prevalent microbial signature in adenomas and adenocarcinomas of the colon [9], also produces high levels of indoles from tryptophan metabolism. The crucial role of tryptophan relies on its ability to transform products from different dietary sources, depending on whether they are processed by beneficial or harmful bacteria [128]. This understanding of the role of the gut microbiome in tryptophan metabolism sheds light on its interaction with fermentable carbohydrates.

### Dietary Carbohydrates

#### Fermentable Carbohydrates in Diabetes and CRC

The consumption of fermentable carbohydrates, such as resistant starch, effectively reduces the byproducts of protein fermentation in the colon, including ammonia and phenols [129]. When fermentable carbohydrates are delivered to the colonic microbiota, there is an increase in carbohydrate fermentation, leading to increased nitrogen utilisation by microbes. This supports their increased mass and creates an acidic environment in the colon, favouring ammonia excretion over absorption into the portal circulation [129, 130]. For example, in an animal model, oncogenic miRNA-expressing rats were simultaneously fed both red meat and high-amylose-resistant starch, which was associated with increased *Ruminococcus bromii*, Bifidobacteriales, Turicibacteraceae, and Lactobacillacea [131]. This diet led to a shift in gut metabolism from primarily protein fermentation to a combination of protein and carbohydrate fermentation [131]. The fascinating process of gut fermentation from Brassicaceae family plants, which are also rich in tryptophan and fermentable carbohydrates, induces microbial metabolites against colorectal tumorigenesis, such as 3-3-diindolylmethane (DIM), Indole-3-carbinol (I3C), Indole-3-acetonitrile (I3ACN) and Indole[3,2b] carbazole (ICZ). These bacteria are derived from health-promoting bacteria, such as *Lactobacillus plantarum* and *Lactobacillus gallinarum* [128, 132, 133]. These findings suggest that improving the quality of carbohydrates, including fibre, fermented by the gut microbiome, enhances the microbiome’s ability to produce anticarcinogenic metabolites in addition to fermentable protein.

### FODMAPs

FODMAPs, which are fermentable oligosaccharides, disaccharides, monosaccharides, and polyols, are a group of short-chain carbohydrates, including galactooligosaccharides (GOS), fructans, lactose, excess fructose, mannitol and sorbitol, which are prevalent in natural foods. Low-FODMAP foods generally have a high glycemic index of easily digested and easily absorbed simple carbohydrates. A high FODMAP diet has decreased subjective hunger and energy intake ratings in clinical and free-living settings [134, 135]. However, the FODMAP content varies across geographical regions, and what is considered “high” depends on the local diet [116, 136]. Eating “excessive” amounts of high FODMAP content can lead to reduced appetite and increased early satiety, fullness, bloating, and flatulence [137]. Few clinical studies have examined the effects of high- and low-FODMAP diets on colorectal patients. It was found that people experienced more diarrhoea when consuming high-FODMAP-rich foods than when consuming low-FODMAP foods [138]. However, this study did not investigate CRC biomarkers or changes in the gut microbiota. An observational study in a French cohort suggested that total FODMAP intake, especially GOS intake, was associated with increased overall risk of cancer, including CRC [139]. This observation contrasts with randomised controlled trials (RCTs) in other severe gastrointestinal diseases, such as inflammatory bowel diseases. Maintaining an appropriate daily intake of FODMAPs is important for promoting a healthy microbiota and protecting the colonic mucosa, as evidenced by the greater relative abundance of butyrate-producing *Clostridium cluster* XIVa and mucus-associated *Akkermansia muciniphila* in typical Australia diet (24 g per day) than in patients with Crohn’s disease receiving a low FODMAP diet (3 g per day) [140].

Additionally, *Ruminococcus torques* were found to be lower in the high-FODMAP diet group [140]. For studies of individual FODMAPs, an RCT investigating navy beans (rich in GOS and fructans) added to their usual diet revealed an increase in S-methylcysteine, pipecolate, 3-(4-hydroxyphenyl)propionate, N-delta-acetylornithine, S-allylcysteine, and 2,3-dihydroxy-2-methylbutyrate in the intervention group compared with the control group (with no dry bean consumed); these metabolites derived from the diet were shown to play positive roles in the suppression of carcinogenesis [141]. In animal models, supplementation with fructooligosaccharides, collectively known as fructans, under anaerobic conditions significantly increased butyrate production and improved SCFA-mediated protection against CRC [142]. In addition, yogurt (high in lactose) protects the colonic mucosa and maintains microbial diversity, which is associated with a decreased risk of CRC, especially in the proximal colon [143]. Fruits, such as apples (high in excess fructose and sorbitol), are also inversely correlated with a reduced risk of cancer at different anatomical sites [144]. These protective effects of individual FODMAPs have been investigated in the prediabetic cohort [116] via interactions with the microbiome [145].

Consistent with other studies, GOS intake was associated with positive outcomes through changes in bacteria that promote short-chain fatty acid (SCFA) production in patients with diabetes and CRC [146–149], with lower gastrointestinal symptoms than other individual FODMAPs [150]. Diarrhoea is caused mainly by excessive FODMAP content entering the gut and undergoing fermentation by the microbiome [151]. Examining the FODMAP content of the local diet is important for establishing the baseline tolerance for induced gastrointestinal symptoms to prevent additional physiological and psychological stress [152]. However, unlike studies on individual food elements, few studies have investigated FODMAPs in the pretreatment or treatment of colorectal cancer and their interaction with the gut microbiome. Moreover, the interaction of diet and medication on the gut microbiome could be a precision therapy for patients with CRC and a preventative measure against diabetes-related CRC.

### Dietary Fibre

Studies have shown that a diet high in vegetables, fruit, cereal, and seeds is linked to a reduced risk of colorectal cancer. Increasing fibre intake by 5 g per day was associated with an 18% lower risk of CRC-specific mortality [153]. Low dietary fibre intake putatively increases the risk for *Fusobacterium nucleatum*-mediated CRC [154]. In particular, dietary fibre, such as acetate, propionate, and butyrate, which are involved in several anticancer mechanisms, significantly modulates CRC risk via the formation of short-chain fatty acids (SCFAs) by the gut microbiota [155, 156]. SCFAs, particularly butyrate, improve intestinal barrier function by reducing permeability and increasing the expression of tight junction proteins such as claudin-1 and zonula occludens-1 [157].

First, butyrate suppresses the activation of the nuclear factor-kappa B noncanonical pathway, which is a transcription factor that regulates the expression of inflammatory genes, such as IL-6, cyclooxygenase-2, and inducible nitric oxide synthase (iNOS) [158]. Second, it has anti-inflammatory effects by upregulating the expression of peroxisome proliferator-activated receptor-γ (PPARγ) in colonic epithelial cells [159]. Third, it increases mucin synthesis and inhibits mucin degradation by the gut microbiome, which acts as a protective deterrent in the gut [160]. Finally, all SCFAs interact with immune cells via G protein-coupled receptors and play an immune surveillance role [156]. Furthermore, secondary bile acids, such as deoxycholic and lithocholic acids, induce tumorigenesis in the bowel, while gut bacteria are unable to regulate the bile acid pool via complex microbial biotransformation [161, 162]. Increased fibre intake can lower the colonic pH, reduce bile acid solubility, and improve mineral absorption by regulating the conversion of secondary bile acids to primary bile acids [163].

Different dietary fibres and supplements affect whole-gut transit time, visceral hypersensitivity, and motility differently [164]. A study using innovative SmartPill electronic capsule technology revealed that consuming 9 g of wheat bran reduced the whole-gut and colonic transit times by 9 and 11 h, respectively [165]. Wheat bran contains dietary fibre but is also rich in other fermentable carbohydrates. The inclusion of highly fermentable carbohydrates, in addition to oligosaccharides, in dietary fibre is recommended to increase the fermentation of SCFAs by the gut microbiota, including *Firmicutes*,* Clostridium, Propionibacterium*,* Bacteroides*,* Bifidobacterium*, and *Lactobacillus* spp. [166, 167], which are beneficial to gut health. These effects have been linked to increased postprandial expression of numerous gut peptide hormones, most notably glucagon-like peptide-1 (GLP-1) [168], which play crucial roles in improving the glycemic response and insulin secretion [169, 170]. Highly fermentable carbohydrates include short-chain fermentable carbohydrates and indigestible or poorly digestible fibre. These substances can increase the fermentation process in the colon and are linked to a decrease in blood sugar levels by improving the functioning of beta cells [171].

### Other Nutritional Elements

#### Calcium

Furthermore, FODMAPs are abundant in plant-based diets and contain essential nutrients such as minerals. Studies have demonstrated a connection between high calcium intake and a decreased risk of CRC, as well as its association with IGF-I [172, 173]. Calcium is hypothesised to exert its anti-neoplastic effects through mechanisms such as the binding of secondary bile acids and free fatty acids in the colon, thereby reducing the exposure of epithelial cells to their damaging effects [174, 175]. Another potential mechanism involves the calcium-sensing receptor (CASR), which is abundantly expressed in the normal colonic epithelium. High calcium intake is significantly associated with a reduced occurrence of CASR-positive tumors [176].

Changes in the distribution of beta diversity and gut fermentation observed in patients with diabetes and CRC suggest that dietary components may regulate microbial metabolism and dynamic equilibrium. According to the above evidence, increasing the consumption of fermentable carbohydrates and decreasing the intake of fermentable protein are generally recommended as a cornerstone of dietary treatment for CRC are generally recommended. This dietary approach can help restore the presence of beneficial bacteria such as *Bifidobacterium*,* Akkermansia*,* and Clostridium* Cluster XIVa and reduce *Bilophila wadsworthia* [177]. This approach ensures nutritional adequacy for individuals with diabetes and CRC and a holistic connection with diabetic drugs (e.g., metformin) in the treatment of CRC predisposed in patients with diabetes by reshaping the gut microbiome [178, 179]. The integration of molecular pathological epidemiology linking diet, microbiome, and tumor microenvironment is essential to explore the dynamic interplay among these factors and develop targeted cancer therapies. However, phenotypic variation, strain specificity, heterogeneity in host responses, and other lifestyle factors can limit the interpersonal variability. Consequently, future interventional studies, including investigation of systemic inflammation, biomarkers and the gut microbiota, are necessary to confirm the effects of these dietary manipulations.

## Conclusion

The gut microbiome should be considered a factor in determining the health of the colon and the body’s ability to obtain necessary nutrients in patients with colorectal cancer. Increasing the intake of fermentable carbohydrates and reducing fermentable proteins could change the microbiome and lead to a shift towards carbohydrate metabolism. This change may help reduce the adverse effects of colorectal cancer in patients with diabetes. More clinical trials are needed to explore the potential of dietary therapy for treating CRC in patients with diabetes.

## Key References

- Murphy, N.; Song, M.; Papadimitriou, N.; Carreras-Torres, R.; Langenberg, C.; Martin, R.M.; Tsilidis, K.K.; Barroso, I.; Chen, J.; Frayling, T.M. Associations between glycemic traits and colorectal cancer: a Mendelian randomization analysis. *JNCI: Journal of the National Cancer Institute* 2022, 114, 740-752.
-
  - This study utilized Mendelian randomization (MR) to assess the causal effects of glycemic traits and fasting insulin levels in patients with type 2 diabetes and colorectal cancer. The findings suggest that pharmacological or lifestyle interventions aimed at lowering circulating insulin levels could be beneficial for preventing the development of colorectal tumors.
- Lawler, T.; Walts, Z.L.; Steinwandel, M.; Lipworth, L.; Murff, H.J.; Zheng, W.; Andersen, S.W. Type 2 Diabetes and colorectal cancer risk. *JAMA Network Open* 2023, 6, e2343333-e2343333
-
  - This study involved 80,193 patients with gastrointestinal cancers across eight European and Asian countries, revealing a high prevalence of diabetes mellitus among digestive tract cancer patients, particularly in those with colorectal cancer.
- Zhong, Y.; Zhu, Y.; Li, Q.; Wang, F.; Ge, X.; Zhou, G.; Miao, L. Association between Mediterranean diet adherence and colorectal cancer: a dose-response meta-analysis. The American journal of clinical nutrition 2020, 111, 1214-1225.
-
  - In this dose-response meta-analysis of Mediterranean diet adherence and colorectal cancer, adherence to the Mediterranean diet was found to be inversely associated with the incidence of colorectal cancer.

## Data Availability

No datasets were generated or analysed during the current study.

## Abbreviations

CRC: Colorectal cancer
IGF: Insulin-like growth factor 1
PI3K: Phosphatidylinositol-4,5-bisphosphonate 3-kinase
FASN: Fatty acid synthase
IGFBP-3: Insulin-like growth factor binding protein 3
GI: Glycaemic index
BCFAs: Branched-chain fatty acids
AAs: Amino acids
HCAs: Heterocyclic aromatic amines
NOCs: N-nitroso compounds
ROS: Reactive oxygen species
LPOs: Lipid peroxidation products
PAHs: Polycyclic aromatic hydrocarbons
COX-2: Cyclooxygenase-2
H_2_S: Hydrogen sulfide
CBS: Cystathionine β-synthase
CSE: Cystathionine γ-layse
3-MST: 3-Mercaptopyruvate sulfurtransferase
LADA: Latent autoimmune diabetes in adults
IDO1: Indoleamine 2,3-dioxygenase 1
DIM: 3-3-diindolylmethane
I3C: Indole-3-carbinol
I3ACN: Indole-3-acetonitril
ICZ: Indole[3,2b] carbazole
SCFAs: Short-chain fatty acids
iNOS: Inducible nitric oxide synthase
GLP-1: Glucagon-like peptide-1
GOS: Galactooligosaccharides
FODMAPs: Fermentable oligosaccharides, disaccharides, monosaccharides, and polyols
EPA-FFA: Eicosapentaenoic acid-free fatty acid
CASR: Calcium-sensing receptor
NOX: NADPH oxidase
